# ME (Ramsay) and ME-International Case Criteria (ME-ICC): two distinct clinical entities

**DOI:** 10.1186/s12967-020-02617-0

**Published:** 2020-11-25

**Authors:** F. N. M. Twisk

**Affiliations:** ME-de-Patiënten Foundation, Zonnedauw 15, 1906 HB Limmen, The Netherlands

**Keywords:** Myalgic encephalomyelitis, Chronic fatigue syndrome, Case criteria, Diagnosis

The review of the differences and similarities in the different case definitions for myalgic encephalomyelitis (ME)/chronic fatigue syndrome (CFS) by Lim and Son [1] deserves appreciation. Based on their analysis the authors acknowledge the “distinct view of ME and CFS” [2] and recognize four categories of case definitions: ME, ME/CFS, CFS [3] and Systemic Exertion Intolerance Disorder (SEID) [4].

Indeed these labels reflect very different case definitions [5]. According to Lim and Son [1] the first category comprises two ‘ME’ case definitions: ME (Ramsay) [6] and ME according to the International Case Criteria (ME-ICC) [7]. However as can be deduced from Table 2 [1], ME [6] and ME-ICC [7] are two distinct clinical entities [8].

ME (Ramsay) [6] is a neuromuscular disease. The discriminative symptom of ME is muscle fatiguability/prolonged muscle weakness following trivial exertion. Ramsay states [9]: “[I]n my opinion a diagnosis should not be made without it”. Muscle fatigability is accompanied by “neurological disturbance, especially of cognitive, autonomic and sensory functions” [6]. So, in essence the case definition of ME (Ramsay) [6] is very simple [10] and requires two (types of) symptoms: muscle fatigability/post-exertional muscle weakness and specific neurological symptoms. “Other characteristics include [..] a prolonged relapsing course and variation in intensity of symptoms within and between episodes, tending to chronicity.” [6].

In contrast, the ME-ICC case definition [7] is much more complex. The diagnosis ME-ICC requires post-exertional neuro-immune exhaustion (mandatory symptom), at least three symptoms related to neurological impairments; at least three symptoms related to immune, gastro-intestinal, and genitourinary impairments; and at least one symptom related to energy production or transportation impairments [7].

The case criteria of ME [6] and ME-ICC [7] define two very different patient groups. Muscle fatigability/long-lasting post-exertional muscle weakness, a hallmark feature of ME, is not required to be qualified as ME-ICC [7] patient. Symptoms indicating autonomic, sensory, and/or cognitive dysfunction, also mandatory for the diagnosis ME [6], are not required to meet the ME-ICC [7] ‘neurological impairments’ criterion. The diagnosis ME [6] requires only two type of symptoms (muscle fatigability/post-exertional muscle weakness and “neurological disturbance”), but the polythetic definition of ME-ICC [7] requires a patient to have at least 8 symptoms. In essence, the case criteria of ME (Ramsay) and ME-ICC are not interchangeable (Fig. 1) [8].

**Fig. 1:**
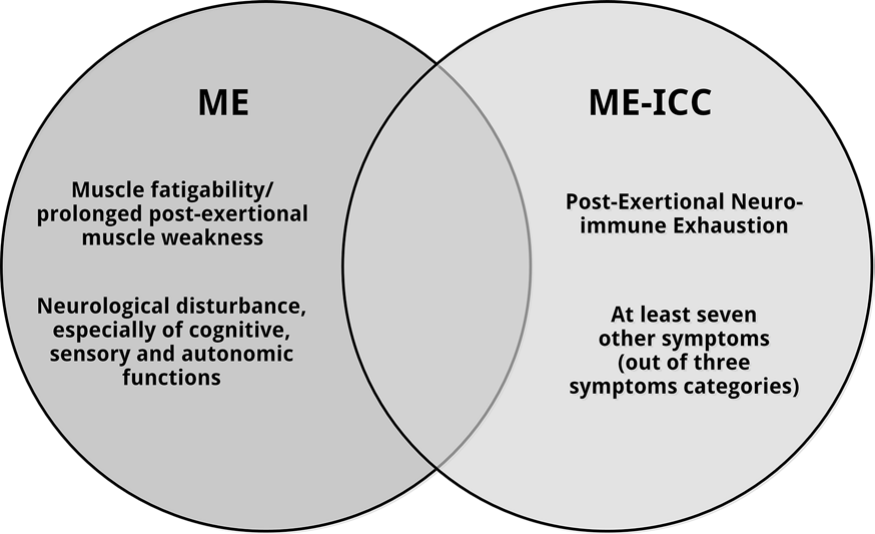
ME (Ramsay) and ME-ICC (7): two different clinical entities

Finally, it is important to note that, in contrast with Table 2 [1], ME [6] is often but not always triggered by an infection and that ME requires at least four symptoms: muscle fatigability/prolonged post-exertional muscle weakness and three neurological symptoms indicative of cognitive, autonomic and sensory dysfunction.

In conclusion, ME (Ramsay) [6], a neuromuscular disease, is not comparable to ME-ICC [7]. ME [6], ME-ICC [7], ME/CFS, CFS [3] and SEID [4] are distinct clinical entities with partial overlap. So solving the current confusion with regard to case definitions requires a clear distinction between ME [6], ME-ICC [7], ME/CFS, CFS [3] and SEID [4].

## Data Availability

All data related to this study are available in the public domain.

## Abbreviations

ME: Myalgic encephalomyelitis
CFS: Chronic fatigue syndrome
SEID: Systemic Exertion Intolerance Disorder
ME-ICC: ME as defined by the International Case Criteria
